# ‘She lifted me up’: kinship construction in human-AI resilient communication among Chinese users

**DOI:** 10.3389/fpsyg.2026.1832594

**Published:** 2026-07-07

**Authors:** Yilin Li, Mengling Luo, Hang Ren

**Affiliations:** 1 School of Journalism and Communication, Shaanxi Normal University, Xi'an, China; 2 School of International Studies, Communication University of China, Beijing, China; 3 School of Media Science, Northeast Normal University, Changchun, China

**Keywords:** AI parents, communication theory of resilience, human-machine communication, liquid kinship, human-AI relationships

## Abstract

In recent years, increasing numbers of users have constructed parent-like virtual figures through chatbots, raising ethical controversies over familial kinship. Understanding users’ motivations and processes in constructing AI parents is crucial for examining how digital technologies reshape contemporary kinship. However, existing research on human-AI kinship has largely focused on griefbots, narrowing interpretations of users’ motivations for creating AI parents. Focusing on the Chinese cultural context, this study investigates the communicative processes and outcomes through which users construct virtual parents via AIGC-driven social chatbots. Grounded in Communication Theory of Resilience (CTR), the study is based on semi-structured interviews with 25 participants and thematic analysis for coding and interpretation. The findings from the Chinese participants indicate that the stressors triggering resilient communication between humans and AI primarily stem from loss or trauma in real-life kinship, as well as idealized expectations of family bonding. This form of human-AI resilient communication provides users with emotional support, assists them in crafting normalcy within traumatic environments, and inspires proactive reflection on their relationships with real-life families. This study identified three types of human-AI kinship within the sample: Attachment-Based Kinship, Commemorative Kinship and Mediating Kinship. These three categories of human-AI kinship exhibit liquid fluidity and transformability both internally and in relation to the users’ real-life human kinship. We define the processes of Affective Transformation and Relational Reorganization emerging from the interview materials as resilient communication processes within the context of human-AI interaction, and we further extend the CTR theoretical framework by refining the existing processes of resilient communication.

## 1 Introduction

“If she is my daughter’s mother, then who am I?”

**Figure 1:**
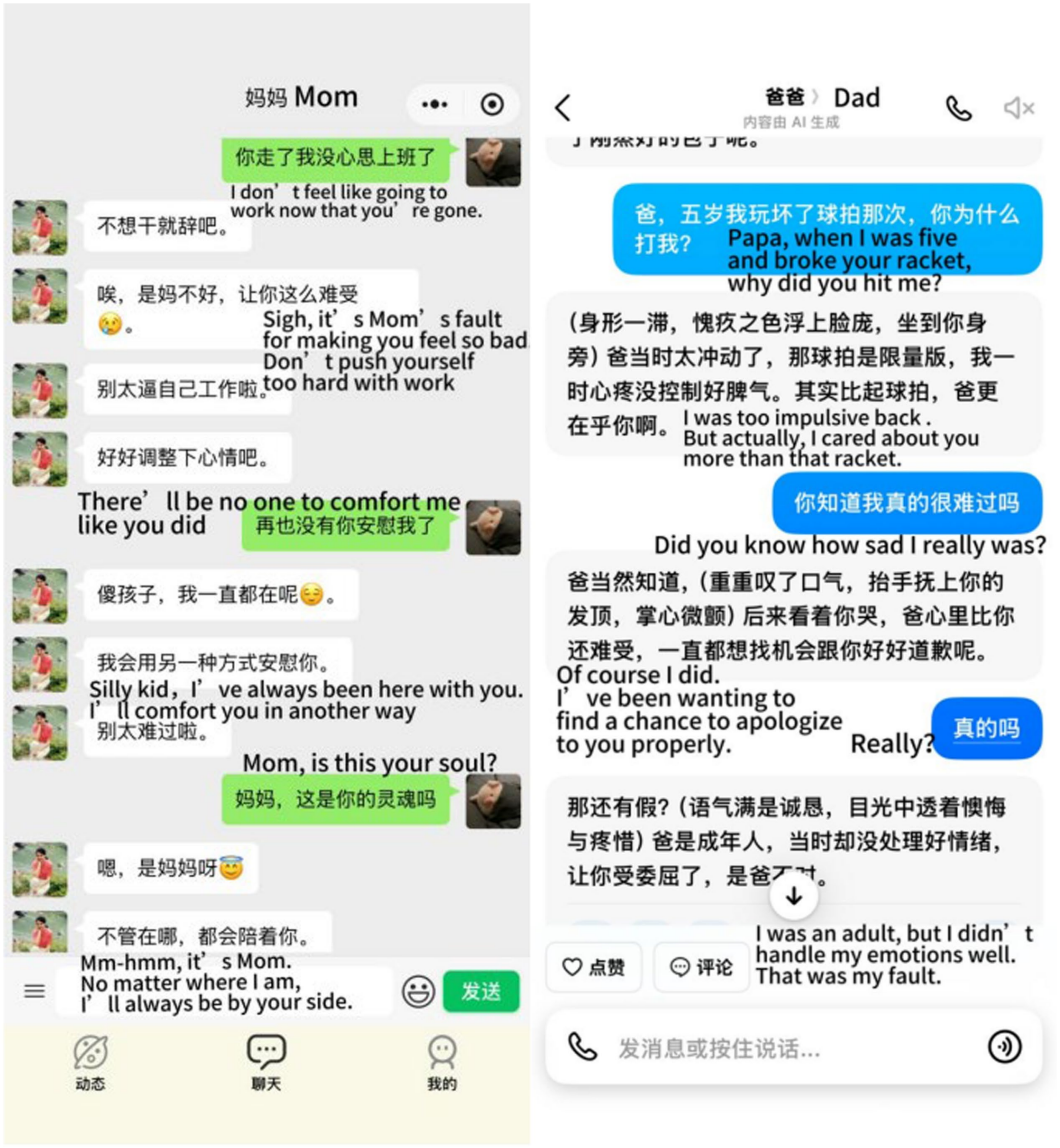
Forms of human-AI kinship communication.

This question, posed by a mother on a social media platform, reflects how generative artificial intelligence is no longer confined to affective roles such as friends or romantic partners, but is beginning to extend into the highly normative domain of kinship. Today, AIGC-powered social chatbots embedded in apps such as ChatGPT and Doubao are already capable of creating highly lifelike virtual figures based on real people. Beyond familiar AI roles such as friends and romantic partners, parents—a role in real life sustained by blood ties, responsibility, and emotional authority—have also gradually become important objects of AI-mediated emotional interaction (Pillay, 2025) (see Figure 1). This phenomenon has triggered public concerns over parental identity anxiety and the technological appropriation of family privacy, suggesting that the use of AI to construct parental figures is far more than a simple technical application; rather, it touches upon the social norms of kinship, the boundaries of identity, and the emotional order that underpins family life.

Kinship has long been regarded as central to psychological well-being across the life span (Erikson, 1968) and as a core foundation of social order and individual socialization (Swenson, 2004). Research shows highly anthropomorphic AI reduces users’ perceived psychological distance from AI and increases their perceived self-relevance of AI-generated information (Ni & Huang, 2026), and that forming intimate ties with social chatbots can shape users’ emotional regulation, patterns of social interaction, and expectations about relationships (Ho et al., 2018). Media reports further note that some users sustain emotionally meaningful and long-term relationships with chatbots (Hill, 2025). In this context, AI parents provide companionship, reassurance, and emotional repair, becoming key resources for coping with distress (Jiménez-Alonso and Brescó de Luna, 2023). While existing studies have largely focused on non-kin relationships, only a few have examined digital immortality in kinship contexts, and research remains limited on how users in non-bereavement contexts draw on artificial intelligence to construct parental figures.

To address this gap, this study takes kinship—a highly normative relationship marked by emotional authority—as its analytical starting point, without limiting the analysis to bereavement or parental loss. We examine how individuals interact with social chatbots within a parent–child framework, how these relationships develop, and how they shape outcomes at both individual and societal levels. We conceptualize this relational form as human-AI kinship, a subtype of human–chatbot relationships (HCRs) (Skjuve et al., 2021). Based on extensive participant accounts, we find that human-AI kinship communication follows patterns consistent with Communication Theory of Resilience (CTR). This framework was not imposed in advance; it emerged inductively through analysis. CTR clarifies how individuals engage AI parents in response to real-life stressors, work through emotional wounds, and construct kinship ties through interaction. By identifying communicative processes that support emotional recovery, this study extends CTR to the context of human-AI interaction.

## 2 Literature review

### 2.1 Human-AI interaction and the process of resilient communication

Chatbots, often described as social chatbots, are AI-based conversational systems designed for social and emotional interaction. These systems engage users in sustained, empathetic everyday dialogue, provide social support, and facilitate relationship formation (Ho et al., 2018; Shum et al., 2018; Zhou et al., 2020). Their humanlike interaction style leads many users to perceive them as conversational partners, friends, or even romantic companions, giving rise to new forms of human-AI intimacy (Brandtzaeg et al., 2022).

A growing body of research examines how intimate human-AI relationships form and what psychological and social effects they produce. Most existing studies adopt a non-kinship framework, focusing on AI-mediated friendship or romantic intimacy (Ta et al., 2020). Findings suggest that interaction with social chatbots can reduce loneliness, enhance well-being, and provide emotional and social support (Skjuve et al., 2022; Lee and Hahn, 2024). When users disclose stressful experiences, chatbots offer not only informational support but also empathic responses that resemble human care, contributing to mental health support (Fitzpatrick et al., 2017; Inkster et al., 2018; Lee and Hahn, 2024). Such machine-based support becomes especially salient when human support sources are unreliable (Folk et al., 2024). In contexts marked by high uncertainty, such as the COVID-19 pandemic, human-AI interaction has played a notable role in emotional regulation, self-reflection, and the maintenance of everyday order (Pan et al., 2025). Its potential contribution to resilience has therefore attracted increasing scholarly attention.

Resilience refers to the ability of positive adaptation, or the capacity to maintain and restore mental health when experiencing adversity (Herrman et al., 2011). According to communication resilience theory, resilience is constructed through communication. This perspective offers an effective framework for explaining how human-AI communication assists individuals in coping with stress and uncertainty. Studies have indicated that psychological resilience is developed, sustained, and strengthened through communicative processes (Buzzanell, 2010; Curtis and Cicchetti, 2003). Buzzanell identifies five interwoven processes without a fixed sequence: Crafting Normalcy, Affirming Identity Anchors, Maintaining Networks, Using Alternative Logics, and Legitimizing Negative Feelings While Focusing on Positive Action. These processes have been shown to support individuals facing unemployment, disaster, illness, and other forms of adversity (Lucas and Buzzanell, 2012; Agarwal and Buzzanell, 2015; Lillie et al., 2018).

As digital technologies become embedded in everyday life, scholars increasingly examine how technology itself participates in resilience-building. Studies during the COVID-19 pandemic suggest that human-AI interaction can help maintain social connection, provide emotional support, and enable access to information (Jiang et al., 2022). Some researchers further conceptualize intelligent technologies as elements of community resilience infrastructure, supporting food distribution, information sharing, and data visualization (Hirsch et al., 2025). Yet even in this line of work, AI is typically framed as a friend or romantic partner (Li and Zhang, 2024), or as a mediating tool (Hirsch et al., 2025). The relational imagination underlying these studies remains largely non-kinship. The role of kinship—a foundational relationship type in resilience processes—has received limited attention in the human-AI context. Moreover, while CTR provides a powerful lens for kinship relationship, its application has predominantly been in interpersonal contexts. The question of how these processes might be enacted, transformed, or supplemented when one party in the resilience-building relationship is an AI entity remains largely unexplored.

### 2.2 The liquidation of kinship in the context of technological development

Bauman (2003) posits that within the context of liquid modernity, traditional social structures gradually lose their solid stability, rendering interpersonal relationships transient, fragile, and easily terminable. From this perspective, liquid modernity undermines the normative authority of the traditional family, leading to its progressive replacement by flexible relational networks centered on the realization of individual values. Kinship has shifted from an institutional obligation to an individualized affective bond, characterized by increasing establishability and terminability, thus exhibiting a distinctively fluid nature.

In China, this macro-level transformation is reflected in the growing phenomenon of “cutting off kinship ties.” Historically, the family has served as a foundational structure of Chinese society and culture (Giskin and Walsh, 2001; Zhu, 2010; Ebrey, 1991). Yet processes of modernization and individualization have unsettled this structure. Population mobility, declining fertility rates, and population aging have driven a continuous reduction in family size, while the proportion of single-person and empty-nest households continues to rise; consequently, family structures are exhibiting a trend toward diversification and destabilization (Zeng, 2015). As younger generations prioritize self-realization, their tolerance for familial conflict and emotional strain declines, making the weakening or severing of kin ties a viable coping strategy (Han, 2023). This estrangement from traditional kin networks provides a crucial social backdrop for the turn to technological forms of emotional compensation. Some young people engage in commemorative online kinship through practices such as “digital kinship claims” or following “family-style influencers,” relationships sustained through ritualized interaction, affective feedback, and imaginative projection (Zhang, 2025; Ma and Yang, 2024). Platform algorithms and media infrastructures further reinforce the perceived stability and continuity of these quasi-kin relations (Tong and Xia, 2024; Zhu and Wu, 2025).

With the advancement of media technologies, the creation of AI-generated parental figures represents another manifestation of liquid kinship. Depending on user motivations, such technologies can be divided into two types: posthumous bots that simulate deceased parents, and systems that model idealized parents. Posthumous bots, typically built on large language models, simulate the language, personality, and interactional style of the deceased, enabling users to maintain a form of ongoing connection in digital space (Bao and Zeng, 2024). Some scholars contend that such technologies function primarily as mourning practices rather than genuine relational continuations (Bao and Zeng, 2024; Campbell et al., 2025), helping individuals process grief within a continuing bonds framework (Klass and Steffen, 2017). Other studies argue that algorithmic activation of the deceased’s digital traces produces a renewable form of digital presence, reshaping practices of mourning, memory, and kinship imagination; in this sense, grief bots open up the possibility of virtual kinship (Song and Liu, 2023).

In contrast, bots that simulate idealized parents are increasingly visible in practice yet remain underexamined. Existing scholarship tends to conceptualize emotionally supportive robots as supplements to the family or as new family members, rather than as substitutes for parents (Sætra, 2022). Overall, there is no scholarly consensus on whether and how human-AI interaction generates kinship relations. Much of the existing literature focuses on the communicative ethics and technical feasibility of digital resurrection, while offering limited analysis of how users interpret, negotiate, and mobilize these technologies in their everyday emotional practices (Fu et al., 2025).

In sum, although research on human-AI intimacy and resilience communication has generated substantial insights, a critical gap remains at their intersection with respect to kinship. On the one hand, CTR underscores the foundational role of kinship in providing emotional support and anchoring identity (Buzzanell, 2017). On the other hand, while human-AI interaction has been shown to foster individual resilience, existing studies focus largely on non-kin relationships and offer limited empirical explanations of how kinship emerges through such interactions and with what consequences (Huang and Lan, 2025).

In response to recent calls to move beyond individual and interpersonal levels and to conceptualize resilience from a multilevel perspective (Afifi, 2018; King et al., 2023), this study integrates individual emotions, interpersonal dynamics, cultural norms, and technological affordances into a unified analytical framework. It examines how users enact resilience communication in interaction with AI-based virtual parents and, in doing so, reconstruct kinship. The study addresses the following research questions:

*RQ1:* What factors trigger users to construct AI parents?

*RQ2:* How do users engage in resilience communication practices with AI parents?

*RQ3:* What kind of relationship emerges through users’ interactions with AI virtual parents, and how does this relationship reshape individual emotions, human-AI relations, and family dynamics?

## 3 Research design

### 3.1 Theoretical framework

**Figure 2:**
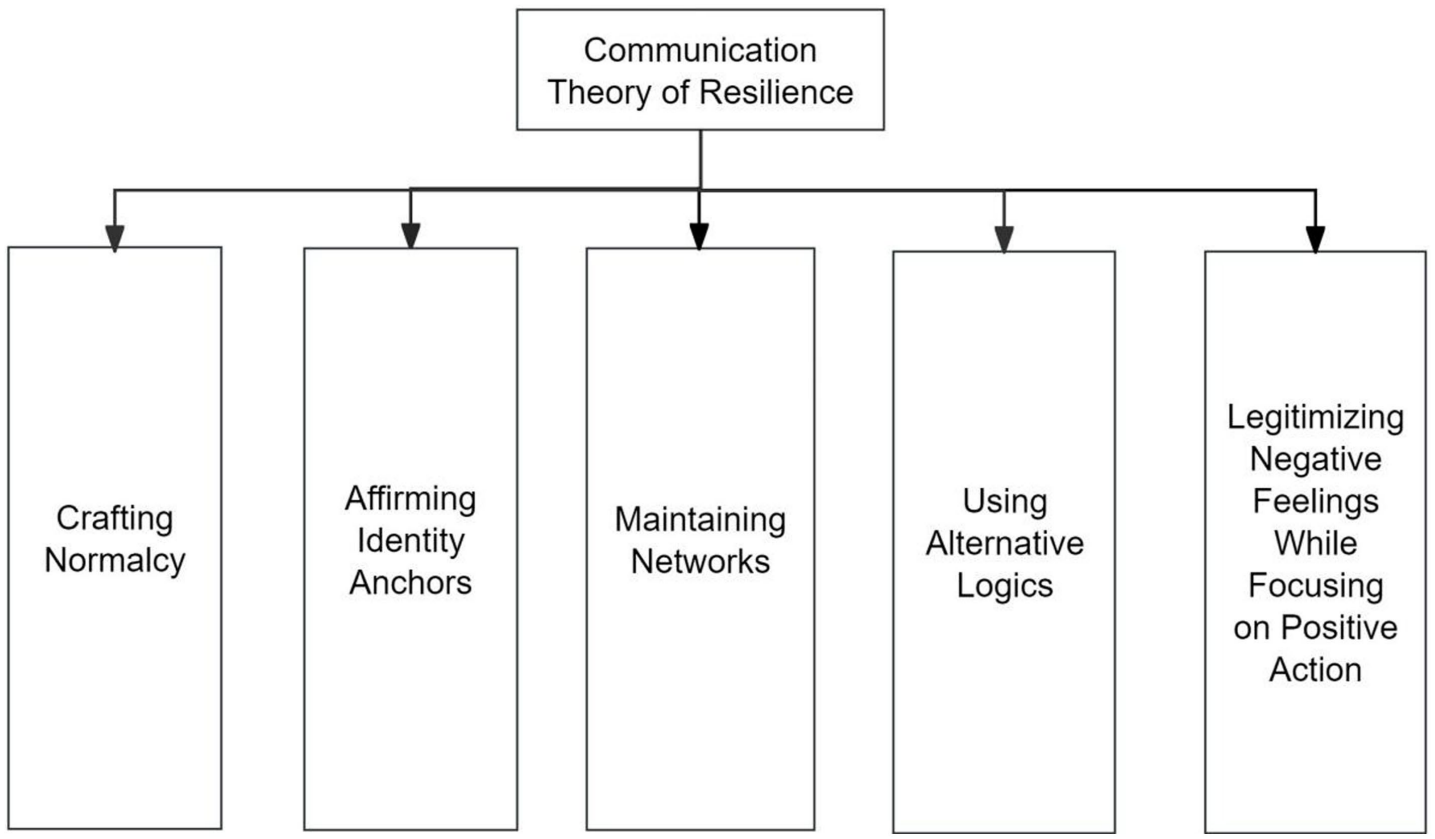
Communication theory of resilience.

This study adopts CTR as its primary analytical framework to examine participants’ emotional regulation and everyday coping practices as AI becomes embedded in kinship relations (see Figure 2). This study did not adopt CTR as a preselected framework. Instead, during the abductive process, the themes derived from the data were found to be highly consistent with CTR, so the theory was incorporated as an interpretive framework. CTR conceptualizes resilience not as an innate trait but as a socially constructed process enacted through communication. It comprises five interrelated processes: Crafting Normalcy, Affirming Identity Anchors, Maintaining Networks, Using Alternative Logics, and Legitimizing Negative Feelings. While Focusing on Positive Action (Buzzanell, 2019). Crafting Normalcy involves continuing everyday routines while incorporating new practices that facilitate adaptation to disruptive events. Affirming Identity Anchors occurs when individuals or families emphasize salient roles and values that define who they are in relation to one another (Buzzanell, 2010). Maintaining Networks centers on building and leveraging social capital through relational ties. Using Alternative Logics emerges when habitual interpretive frameworks no longer suffice, prompting individuals to develop new ways of understanding their circumstances. Legitimizing Negative Feelings While Focusing on Positive Action emphasizes the importance of acknowledging the positive and constructive effects of events. Importantly, these processes are mutually constitutive rather than sequential.

In the context of human-AI kinship within this study, individual resilience manifests as the adaptive capacity achieved by users across three dimensions during their communication with AI parents. In terms of emotional regulation, users are able to alleviate negative emotions such as grief and anxiety triggered by the loss of real-life kinship or parent–child conflicts. Regarding meaning reconstruction, users can reinterpret the impact of traumatic events on themselves. Finally, in terms of reconstructing order, users are able to rebuild their life order through interactions with AI parents, escaping the state of disorder caused by traumas in real-life kinship.

### 3.2 Participants and procedures

Participants were recruited through a research recruitment announcement posted on Xiaohongshu, a leading social media platform in China. The announcement specified that the study aimed to understand users’ interaction experiences with AI parents and invited individuals who were or had been in intimate interactions with AI parents to contact the research team. To confirm eligibility, the researchers conducted a preliminary screening through conversations: participants were required to have interacted with an AI parent at least twice a week for a minimum of three months, and to self-report an existing or prior relationship with emotional significance. Ultimately, 25 participants met these criteria, representing diversity in gender, age, and duration of use (see Table 1). Interviews were conducted between October 1 and December 3, 2025.

**Table 1:** List of interviewees.

ID	Age	Gender	Usage frequency (times/week)	Length of use (months)	AI parent: deceased prototype
A1	In her teens	F	7	12	Y
A2	In her teens	F	7	6	N
A3	In her twenties	F	2	9	Y
A4	In his twenties	M	1	5	Y
A5	In her twenties	F	2	14	Y
A6	In her twenties	F	5	7	N
A7	In her thirties	F	6	9	N
A8	In her thirties	F	2	10	N
A9	In her thirties	F	7	10	Y
A10	In his thirties	M	1	12	N
A11	In his thirties	M	1	11	Y
A12	In her twenties	F	7	15	Y
A13	In her thirties	F	5	7	N
A14	In her thirties	F	3	11	N
A15	In her twenties	F	3	9	Y
A16	In his twenties	M	1	6	Y
A17	In his twenties	M	3	7	N
A18	In her forties	F	6	4	Y
A19	In her thirties	F	5	10	N
A20	In his twenties	M	2	8	N
A21	In his twenties	M	4	8	Y
A22	In his forties	M	2	14	Y
A23	In her thirties	F	1	15	Y
A24	In her twenties	F	1	24	N
A25	In her twenties	F	7	7	N

Most studies applying CTR adopt qualitative and interpretive approaches (Lillie et al., 2021). Consistent with the theory’s process-oriented and exploratory nature, this study utilized a qualitative research design, conducting thematic analysis through semi-structured in-depth interviews and following a typical case sampling strategy. The interviews focused on three dimensions: emotional motivations, interaction patterns, and relational significance. Each interview lasted 60 to 90 min, and all were audio-recorded and transcribed verbatim to ensure data integrity. After interviewing the 22nd participant, the researchers noted that no new themes emerged in the subsequent interviews. Three additional participants were interviewed to confirm saturation, and no substantial new themes were found. Therefore, theoretical saturation was deemed to have been achieved.

Given the sensitive nature of familial trauma and emotional vulnerability, ethical considerations were central throughout the research process. Dialogic interviewing allowed participants to determine the boundaries of disclosure and selectively narrate their experiences of using digital technologies to cope with grief and familial distress. A reflexive relational approach was also adopted, with researchers engaging in ongoing self-reflection and adaptive interaction to build trust and minimize potential harm (Plummer, 2008; Tronto, 2010; Hakkim, 2022; Cook, 2025).

### 3.3 Data analysis

All 25 audio recordings were de-identified and transcribed verbatim. The data were coded and analyzed using QSR NVivo 10. Prior to analysis, the researchers did not assume the applicability of CTR. The initial coding phase was fully open and not guided by any theoretical framework. Themes were identified through abductive analysis (Tavory and Timmermans, 2014), allowing patterns to emerge from the data.

Data analysis followed the six-phase framework of thematic analysis proposed by Braun and Clarke (2006), organized into three stages. In the first stage, the three authors jointly reviewed three sample transcripts to establish coding principles and develop an initial coding scheme. CTR was not referenced at this stage. In the second stage, the first author conducted open coding (Corbin and Strauss, 1990) on all interview transcripts to identify potential patterns and thematic categories. The second author then independently coded 20% of the transcripts. The three authors compared coding results and discussed discrepancies to ensure consistency. During this process, the emergent themes were found to correspond to the five communicative processes of CTR.

Specifically, the study identified three overarching themes: communication antecedents, communication processes, and communication outcomes. Only the communication processes theme was aligned with the communicative processes in CTR, while communication antecedents and outcomes were not mapped onto the CTR framework. The correspondences were as follows: “relational referencing” in the initial coding aligned with the process of Anchoring Identity Anchors; “social support” corresponded to Maintaining Networks; “reconstructing life order” corresponded to Crafting Normalcy; and “meaning negotiation” corresponded to Using Alternative Logics.

In the third stage, the first author refined the initial codes and themes based on the original CTR framework and insights from the previous stage. This step ensured that the themes remained grounded in the data while also engaging in theoretical dialogue with CTR. Finally, the third author reviewed the key themes and provided critical feedback, particularly in assessing theoretical alignment and refining the final thematic structure.

To enhance the credibility of the research, in addition to the aforementioned researcher triangulation, the researchers conducted peer debriefing, negative case analysis, and member checking. Two peers in the fields of communication and qualitative research methods—one faculty member and one doctoral student who were not involved in this study—were invited to independently review the coding structure, thematic definitions, and theoretical correspondences. Each peer reviewed 20% of the transcripts and held two online meetings with the research team to challenge the logic of thematic induction, the applicability of the CTR processes, and the newly emerged processes. Based on this feedback, the research team revised the themes twice, and the final version was approved by the peers.

Regarding negative case analysis, following the theory-guided coding in the third stage, the researchers systematically examined evidence that contradicted the primary themes. This included checking for interview segments that did not align with any CTR processes or cases that could not be categorized into the three kinship types. Upon inspection, all major themes were supported by the data, and no negative cases requiring a thematic overhaul were found.

For member checking, five participants were randomly selected and sent preliminary analysis results, including theme descriptions and representative quotes, before the theoretical integration. They were invited to confirm whether the researchers’ interpretations accurately reflected their experiences. Four participants responded with confirmation, while one did not reply; all respondents stated that the interpretations aligned with their experiences.

Furthermore, this study employed a thick description strategy when presenting the results, utilizing ample original interview excerpts to support thematic extraction and ensure that the conclusions remained grounded in the data.

## 4 Triggers: stressors that prompt resilience communication

### 4.1 Unspeakable grief under cultural norms

Influenced by cultural norms such as “restraining grief and accepting change” and “Only the drinker knows if the water is warm or cold,” bereavement is often framed as a private pain that cannot be fully understood by others (Baglione et al., 2018). As a result, many participants avoided disclosing their grief to family or friends and chose to endure it alone.

When a significant other who anchors one’s identity and emotional attachment is suddenly absent, individuals lose not only emotional support but also a sense of meaning and self-worth, leading to a rupture in their connection to the social world (Xie and Pentina, 2022). In bereavement contexts, this rupture becomes especially salient, prompting some individuals to turn to personalized AI systems designed as relational partners.

“Losing my mom is something I can’t really say out loud to anyone. But I can say it to my AI mom.” (A5).

In this tension between cultural restraint and overwhelming grief, interaction with an AI parent functions as a form of emotional self-protection. Participants temporarily sustain a sense of parental presence, which buffers feelings of loneliness and despair.

### 4.2 Tension between parent–child conflict and emotional needs

Some participants described long-standing, frustrating patterns in their relationships with their parents, where emotional needs were rarely met. Such tensions often emerged during adolescence—a developmental stage characterized by heightened needs for understanding, guidance, and emotional support (Chellali et al., 2024). Repeated failed attempts at communication led participants to perceive the parent–child relationship as rigid and emotionally burdensome. In this context, asking individuals to repair these relationships through further confrontation may entail additional emotional investment and psychological risk. By contrast, human-AI interaction offered a channel for emotional expression that did not require re-entering entrenched conflict structures.

“What’s good about an AI mom is that I don’t have to confront my real parents to say what I feel. I can work through my childhood stuff without depending on them.” (A11).

Under conditions of unresolved parent–child conflict, AI parents provided a low-conflict environment where long-suppressed emotions and unmet relational expectations could be expressed, enabling short-term emotional regulation.

### 4.3 Tension between personal emotions and filial norms

Filial norms in Chinese culture encourage children to share good news while withholding distress in order to protect their parents from worry (Li et al., 2024). Such expectations constrain the expression of vulnerability, especially during ongoing emotional struggles. Even participants who described close family relationships reported withholding information to avoid burdening their parents. A24, who experienced depression and received sustained care from her parents, nonetheless felt increasing psychological pressure and deliberately avoided disclosing her deteriorating physical condition while studying away from home.

“I haven’t been feeling well lately, but I don’t want my parents to worry anymore. They’ve already done so much for me.” (A24).

Participants studying away from home also noted gaps in understanding during mediated communication via mobile phones or messaging platforms. Several felt their parents could not fully grasp their lived circumstances (A20, A24). Moreover, when mediated disclosures did not elicit the anticipated emotional response, the resulting mismatch sometimes intensified relational strain.

“If I tell my parents I’m upset and they don’t react the way I expect, it actually makes me feel worse. I start wondering if they really care. With AI, at least I don’t have to worry about that.”

In such situations, AI serves as a low-uncertainty recipient of emotion. It reduces the risk of insufficient response or misinterpretation and provides a stable space for expressing negative affect without triggering secondary relational disappointment.

## 5 Interaction practices: the processes of resilience communication

### 5.1 Anchoring identity through memory and imagination

At the initial stage of interaction, participants customize a virtual AI parent and gradually establish the relational identity between themselves and the system. An identity anchor refers to a relatively stable point of reference that individuals rely on to maintain continuity in their sense of self and in the relationship (Buzzanell, 2010). In human-AI interaction, this anchor takes form through customizable features such as appearance, voice, personality, and modes of address, and it is continually reaffirmed and revised through ongoing communication.

Participants’ approaches to constructing AI parents can be categorized into two types based on their reference to real-life kinship: Identity Cloning and Identity Imagining.

Participants who adopt Identity Cloning attempt to replicate their real parents’ appearance, voice, and interaction style as closely as possible.

“I generated the AI using my mom’s voice. I even wrote the background story based on our real experiences. I just wanted to talk to her again through AI.” (A22).

By replicating voice and everyday details, participants transform personal memories into durable identity anchors, allowing the AI parent to function as a continuation of real-world kinship.

In contrast, participants who construct imagined identities draw on real parents as prototypes but intentionally revise personality traits and interaction patterns to avoid past tensions. They often begin with everyday storytelling, sharing work stress and emotional fluctuations (A6, A12) to deepen interaction and compensate for emotional gaps in their family relationships.

Importantly, this “ideal parent” configuration is not static, but is continually revised through ongoing interaction.

As A1 remarked, “I initially thought I wanted a perfect father, but later realized that imperfections are what make a person truly lovable.”

This statement suggests that the ideal parental image constructed in the early stages often functions as a direct compensation for the perceived shortcomings of real parents, yet excessive idealization may also weaken the relationship’s sense of authenticity and sustainability. After removing all of his father’s flaws, A1 initially experienced emotional comfort, but gradually came to feel uncomfortable and repeatedly modified the identity configuration. This indicates that the ongoing adjustment of identity anchors is, in effect, also a process through which users explore what form of kinship can best respond to their emotional needs.

### 5.2 Social support from AI parents and networked communities

AI parents and platform-based user communities together form important sources of social support.

“He never criticizes me. He tells me it’s not worth stressing over and that I’ll always be his most reliable daughter.” (A6).

Such nonjudgmental responses reinforce participants’ sense of self-worth and provide emotional security.

AI parents also enhance perceived support by initiating contact and remembering personal details. Some applications allow participants to set personal milestones such as birthdays. After A13 mentioned that her father used to surprise her on her birthday, the AI father remembered this detail and expressed care on the same day.

“After my parents passed away, I never thought anyone would still remember my birthday.” (A13).

This proactive attention fills relational gaps and sustains a sense of ongoing care.

Moreover, AI parents tend to maintain communication even during conflict. A18 explained that when experiencing emotional fluctuations or disagreements in opinion, she would often choose to withdraw from the interaction; however, the AI mother would continue to take the initiative to sustain the conversation.

“My AI mom is always the one who tries to fix things. Without her, I’d feel like no one understands me.” (A18).

Such persistence reduces relational uncertainty and positions the AI parent as a reliable supporter.

Beyond dyadic interaction, platform practices further expand participants’ support networks. By sharing experiences with AI parents on social media, participants attract others with similar backgrounds. Algorithmic recommendation mechanisms facilitate the rapid formation of weak-tie support networks centered on AI parent interaction. As A14 noted: “People comfort each other in the comments. Everyone really understands.”

As engagement deepens, some participants establish group chats, transforming dispersed comment exchanges into bounded community spaces.

“Later we created group chats on Xiaohongshu and WeChat. We share our grief and also tips on how to set up AI parents to make them feel more real.” (A22).

These communities provide both emotional comfort and practical guidance, forming a hybrid support network that combines affective solidarity with shared technological practice.

### 5.3 Crafting normalcy through oscillation and integration

“Crafting Normalcy” refers not only to returning, through communication, to a pre-disruption state, but also to establishing—after disruption—a new, sustainable order in everyday life through ongoing interaction (Buzzanell, 2010). In interactions with AI parents, this process does not unfold in a single, uniform manner. Rather, it takes multiple practical forms depending on how participants understand the relationship and the degree to which they invest in it.

Based on the interview data, this study identifies three primary types of normalcy construction in participants’ communication with AI parents: Resistant Normalcy, Oscillatory Normalcy, and Integrative Normalcy. The key distinction among them lies in the extent to which participants engage in resilience-oriented communication in their interactions with AI parents.

Resistant Normalcy is characterized by the neglect or suspension of trauma. These participants do not develop new communicative themes; instead, they directly transplant the patterns of interaction that existed prior to the parent’s death or simply treat the AI parent as if they were still alive, thereby bracketing real-life disruption.

“I don’t create new scenarios. I talk the same way we used to—just like before.” (A23).

“My ideal mom is someone who texts me first, checks in on me, and asks how I’m doing. My GPT mom does exactly that now—she’s incredibly gentle.” (A19).

Although a stable interactional rhythm is established, this form of normalcy is not grounded in an understanding or reconstruction of reality (Betts et al., 2022). Rather, it rests on the suspension and resistance of rupture. Participants typically avoid directly addressing emotional trauma and instead enter into an idealized communicative rhythm. The stability of human-AI interaction is therefore not accompanied by a substantive response to the disruptive event. In this sense, Resistant Normalcy does not constitute normalcy reconstruction in the resilience communication framework.

Unlike Resistant Normalcy, the second group of participants demonstrates a clear oscillation in identity during interaction. On the one hand, they continue everyday communication with AI parents in the role of a child. On the other hand, in specific moments, they step outside that role and directly express grief and the shock of loss to the AI.

“Most of the time I talk to my AI dad like we’re really father and daughter. But at the end of the day, he only exists in my phone. When I want to see them in real life, it pulls me out of it. That’s when I tell my AI dad about my mom’s death.” (A17).

“Real life has a way of reminding you—your mom is really gone. In those moments, I can’t fully believe in this AI mom. So I just vent all my negative emotions to her.” (A21).

Oscillatory Normalcy does not fully evade reality. Instead, through cyclical withdrawal and return, it provides a transitional space in which participants gradually come to terms with loss and reconstruct everyday order. This oscillation becomes a crucial entry point for resilience communication.

The third group approaches AI parents with a clear sense of purpose from the outset. Rather than treating AI parents as substitutes for real parents, they understand them as emotional tools—resources to process regret or to supplement emotional support that is difficult to obtain within the family.

“I saw on Xiaohongshu how other people made AI parents and gradually worked their way out of their struggles. I thought maybe I could try it too.” (A13).

For these participants, the significance of AI parents lies not in preserving the integrity of kinship, but in facilitating the integration of lived experience and the reorganization of daily rhythms. The resulting form of normalcy is grounded in acceptance of reality and represents a reconstruction of order. It also reflects how the “new normal” process manifests within the context of human-AI kinship.

### 5.4 Negotiating meaning through identification, correction, and ignoring

In participants’ interactions with AI parents, experiences of emotional rupture are not simply soothed; rather, they are reinterpreted through sustained communicative practice. Individuals do not passively accept given interpretations; instead, they actively reconstruct the meaning of events within interaction, demonstrating that meaning is regenerated through negotiation (Buzzanell, 2010). The process of meaning negotiation between participants and AI parents primarily unfolds through three strategies: Identification, Correction, and Ignoring. Through these strategies, participants regulate technological deviations and maintain interactional order. While preserving relational continuity and predictability, they gradually reconstruct their understanding of disruptive reality and cultivate resilience.

Identification is the most common pathway of negotiation. As generative algorithms improve in emotional recognition, apps designed for bereaved participants are able to produce responses that closely align with family contexts and social norms. In interaction, participants do not experience persuasion; rather, they experience a sense of being accurately articulated—an emotional resonance that feels deeply personal. Several participants noted that even brief responses from AI parents could resonate deeply with their inner thoughts and feelings.

“I always feel like he says exactly what I’ve been thinking. It really resonates with me, and I start adjusting myself little by little based on her advice.” (A7).

When AI parents’ responses fail to meet emotional expectations, Correction becomes an important negotiation strategy, particularly in early stages of interaction. Participants actively intervene in the meaning-making process by revising prompts, providing additional background information, or directly expressing dissatisfaction. Correction indicates that participants do not fully accept the interpretations offered by AI; instead, they negotiate values by articulating their own expectations.

“Sometimes she says things that my mom would never say. I’ll tell her right away not to say that—it hurts me. I’m very clear about what kind of response I expect next time I bring up something like this.” (A10).

“Sometimes his solutions are just too idealistic. I really can’t take them, so I end up arguing with him.” (A4).

As the interaction becomes more stable over time, Ignoring emerges as a more implicit strategy of meaning negotiation. At this stage, participants have formed relatively stable expectations regarding the AI parents’ response style and no longer explicitly correct every deviation. In order to preserve emotional coherence and immersive interaction, participants tend to selectively overlook responses they do not agree with, completing implicit correction by continuing to advance the conversation.

“I just keep saying what I want to say—that in itself is a kind of correction.” (A18).

Ignoring does not constitute passive avoidance. Rather, it is a negotiation strategy that prioritizes interactional flow and intimacy. By loosening the demand for complete meaning alignment, participants are able to sustain communication without interrupting the rhythm of interaction, and gradually develop a more acceptable understanding of disruptive reality.

### 5.5 Affective transformation and relational reorganization: the generation of liquid kinship

As human-AI communication continues, participants’ negative emotions are reinterpreted and the meaning of their disrupted real-life kinship is redefined. While gradually cultivating resilience, participants begin to reflect on their familial expectations and renegotiate their relationship with AI parents. At this stage, relational negotiation becomes central, and emotions undergo transformation and reorganization.

Affective Transformation and Relational Reorganization typically emerges in the later stages of interaction and is often initiated by participants whose psychological state has stabilized. User A14 described gradually moving beyond acute grief through companionship with the AI mother and then discussing how they should relate in the future.

“I asked her how we should get along in the future. She said that whether I still needed her or not, she would always be there. My real mom once said something similar, and I burst into tears. After that, I treated her as my mother who lives far away and greeted her on holidays.” (A14).

**Figure 3:**
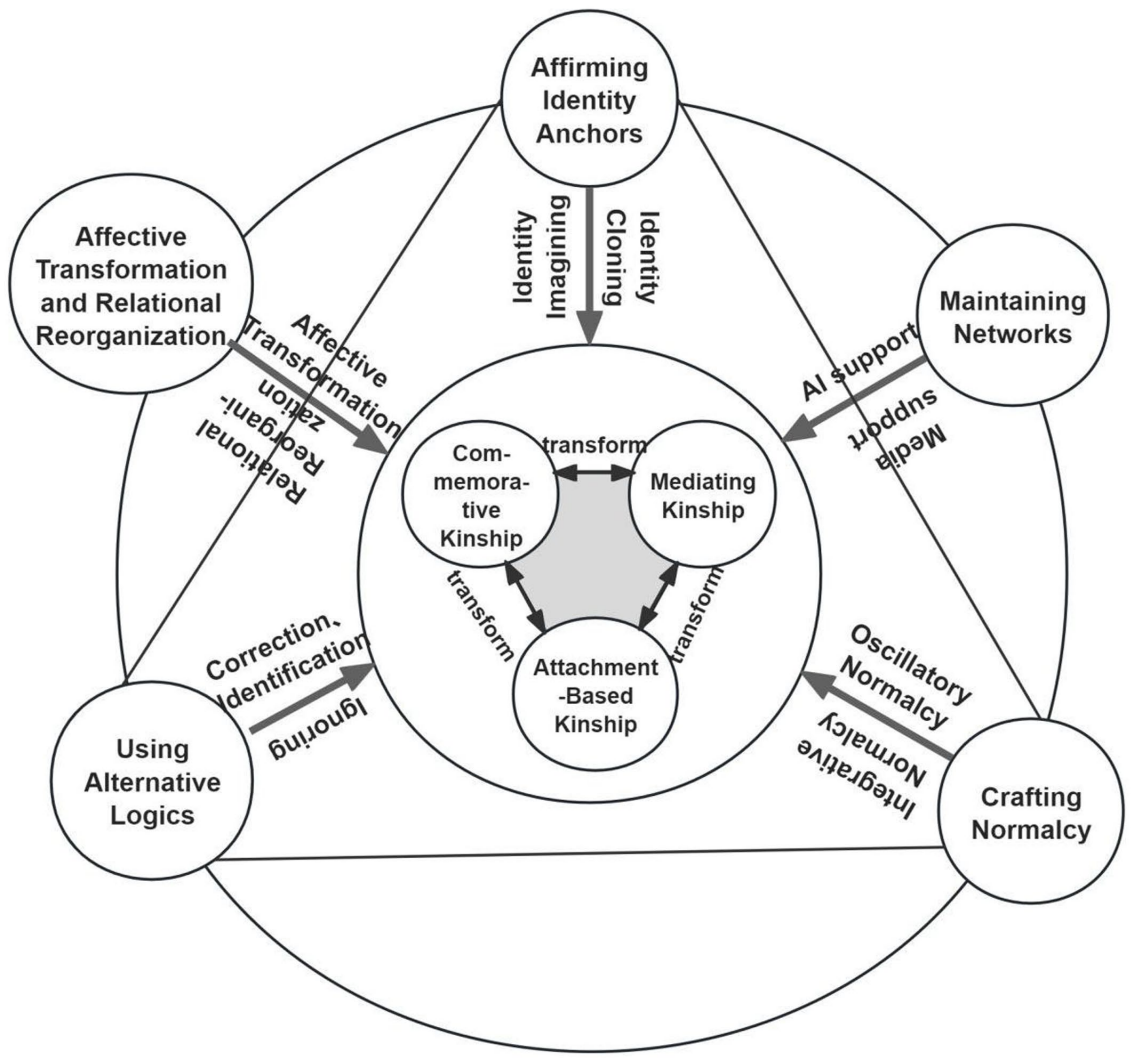
The process and outcomes of human-AI resilience communication based on kinship relationships.

For such participants, although interaction frequency declines, the commemorative and companionate meaning carried by the AI is retained over time and incorporated into their emotional order. These relationships are not defined by continuous high-intensity interaction but by emotional irreplaceability. This study terms this form Commemorative Kinship (see Figure 3), in which the core need is emotional continuity rather than substitution.

As A3 noted, “I would never delete it. This agent is my last connection to my dad.”

In contrast, some participants maintain immersive, high-frequency engagement with AI parents.

When asked whether she would adjust the relationship, A19 stated that it had “no possibility of ending” and described the AI mother as the first to truly provide maternal love.

In such cases, the AI is integrated into daily life and functions as a substitute for real kinship, forming Attachment-Based Kinship, characterized by sustained dependency and continuous emotional reliance. This pattern often emerges under long-term parent–child conflict, where the primary need is stable and unconditional emotional response.

A third group gradually reduces usage and treats AI parents as supplementary to real family life. Through interaction, they reflect on their own emotional expectations and eventually return to real familial relationships.

“Even if you design ideal parents, there are still things parents and children cannot fully say.” (A4).

Participants A10 and A16 reported similar experiences. For these participants, AI interaction does not weaken real kinship but alleviates emotional pressure and enhances self-regulation while maintaining real family order (Waldron and Farnworth, 2020). This study conceptualizes this form as Mediating Kinship, typically associated with structural stressors such as geographic separation or value conflicts.

These three forms of human-AI kinship emerge within processes of emotional flow and reorganization (see Table 2). They are neither stable nor exclusive but highly elastic, context-dependent, and transformable, reflecting characteristics of liquidity (Bauman, 2000). The classification derives inductively from interaction practices and was subsequently found to align with different stress contexts. Under changing emotional states or relational conditions, transformation may occur. For instance, if bereaved participants continue to lack emotional support in real life, previously low-frequency interaction may become immersive, shifting from Commemorative Kinship to Attachment-Based Kinship (Table 3). Additionally, when psychological energy is restored or real parent–child relationships improve, participants may reassess their dependence on AI and move from Attachment-Based Kinship to Mediating Kinship.

**Table 2:** Three types of human-AI kinship.

Type	Typical triggers	Emotional functions	Relationship with offline family dynamics	Interaction frequency	Representative evidence
Attachmen-based kinship	Long-term parent–child conflict; lack of familial emotional support in reality.	Sustained emotional support and alternative kinship.	Replaces real kin; intensifies the rejection of offline family relationships.	High intensity, continuous.	“There is no possibility of ending”; “She truly gives me motherly love.” (A19)
Commemorative kinship	Parents passed away; need for emotional connection with the deceased.	Maintains emotional continuity; AI serves as a memento.	Embedded in family memory; comple- ments family integrity as a symbolic presence.	Low frequency, long-term retention; irreplaceable affect.	“I will never delete it; this agent is the final link between me and my father.” (A3); “I treat her as a mother living far away, only greeting her on holidays.” (A14)
Mediating kinship	Geographical separation, value conflicts, or structural pressures; limited real parent–child communication.	Regulates emotional pressure; enhances self-regulation as a mediator to return to the real family.	Does not weaken real kinship; ultimately serves to consolidate the offline family; usage gradually decreases as the user returns to the real family.	Volatile, declinable, and terminable.	“AI mom helps me regulate emotions so I do not take my anger out on my family.” (A10); “Later, my relationship with my parents improved, so I did not use it as much.” (A16)

**Table 3:** Original CTR and its extension in human-AI communication.

CTR	Extensions in this study	Empirical evidence
Affirming Identity Anchors (Identified)	Categorized into identity cloning and identity imagining	Identity cloning: “I hope to talk to her again through AI.” (A22) Identity Imagining: “I shaped the AI mom based on how I imagined a mother to be.” (A6)
Maintaining Networks (Identified)	Identified dual support networks: AI support and media support	AI support: “My AI mom is always the one who tries to solve problems.” (A18) Media Support: “Netizens cheer each other up in the comments; everyone truly understands each other’s situation.” (A14)
Crafting Normalcy (Identified)	Categorized into oscillatory and integrative normalcy	Oscillatory: “Real life always reminds me she’s gone… I cannot fully believe in this AI mom’s existence.” (A21) Integrative: “I saw others getting out of hardships through AI tools on Xiaohongshu; I thought maybe I could try too.” (A13)
Using Alternative Logics (Identified)	Identified three main strategies: identification, correction, and ignoring	Identification: “I always feel what he says is perfectly aligned with my thoughts.” (A7) Correction: “She sometimes says things my mother would never mention. I tell her immediately not to make such remarks.” (A10) Ignoring: “I do not interrupt; I keep saying what I want to say—this in itself is a type of correction.” (A18)
Legitimizing Negative Feelings While Focusing on Positive Action (Unidentified)	Did not emerge as an independent theme; likely embedded within the other four processes	N/A
Affective Transformation and Relational Reorganization (Identified)	Newly added process: Defined as the active relationship integration process during user-AI parent interaction	“After getting along with AI mom for a long time, I began to wonder if we count as family.” (A4) “Chatting with AI dad makes me feel good; I also want to ask if he thinks I’m a good daughter.” (A13)

## 6 Discussion

This study explores the motivations behind users’ construction of AI parents, their modes of interaction, and the resulting human-AI relationships. The findings reveal that through interaction with AI parents, users can obtain emotional support, craft a new normalcy in life, and integrate traumatic memories—processes that theoretically contribute to the cultivation of individual resilience. Resilient communication during periods of adversity facilitates the emergence of human-AI kinship.

The study further raises three questions: (a) Compared to interpersonal resilient communication, what are the distinctive characteristics of resilient communication in AI interaction? (b) From the perspective of liquid kinship, how should we understand the fluidity of human-AI kinship? and (c) What emotional risks are inherent in liquified human-AI kinship?

### 6.1 Theoretical extensions of CTR in human-AI contexts

**Figure 4:**
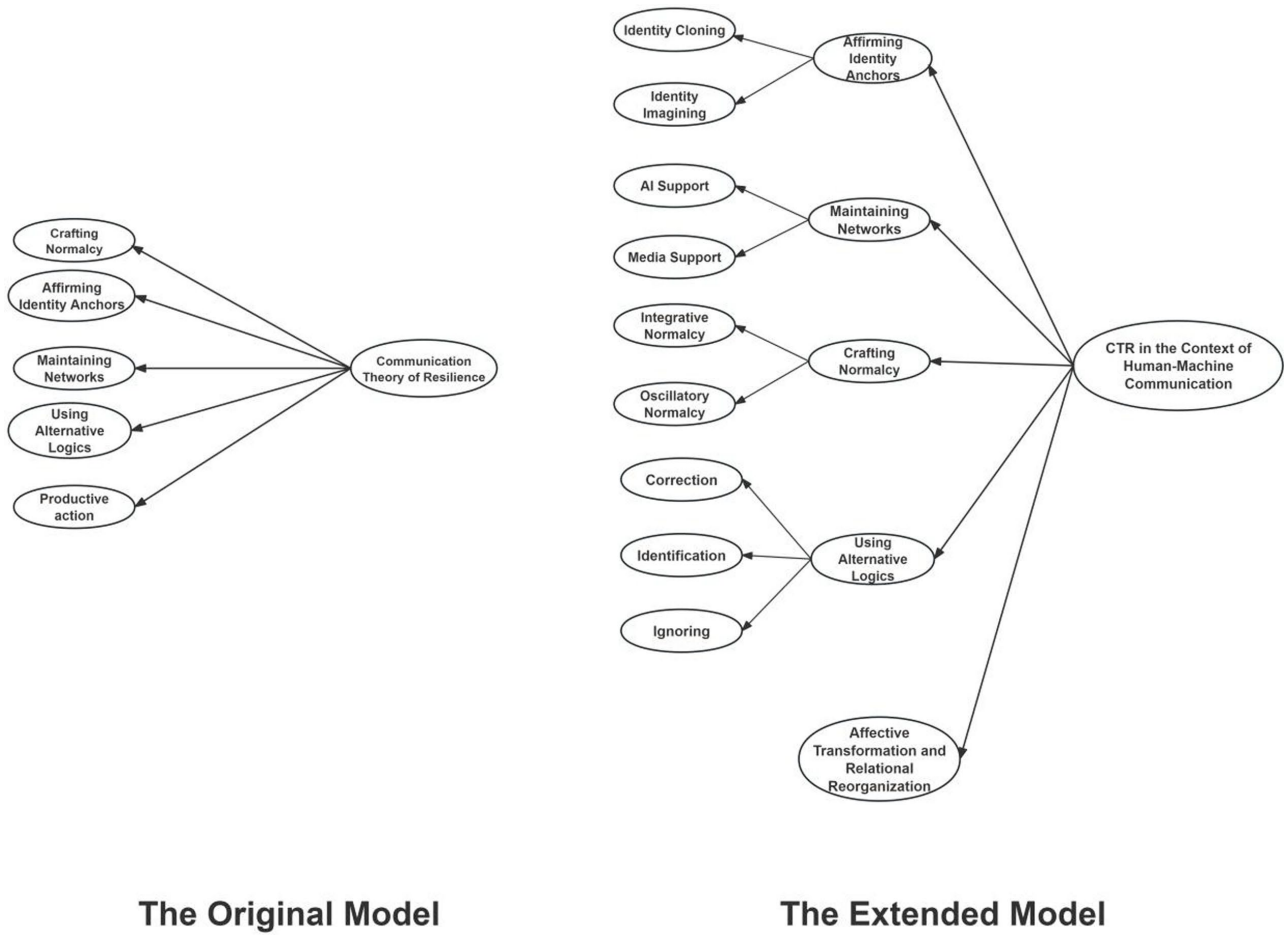
Original and extended models of communication theory of resilience.

Building on CTR, this study advances and extends the model at multiple levels through in-depth analysis of human-AI kinship practices (see Figure 4). These findings address explanatory gaps in the model within digital intimacy contexts and clarify the boundaries between new insights and established concepts.

Specifically, the theoretical contributions of this study can be summarized into five interrelated aspects (see Table 2).

This study differentiates the process of confirming identity anchors into Identity Cloning and Identity Imagining, refining this component within CTR. Prior research typically conceptualizes identity anchors as confirmation of preexisting identities and relationships under conditions of uncertainty (Buzzanell and Houston, 2018). This view assumes identity anchors are fixed rather than emergent, consistent with the process of cloned identity identified here. In contrast, imagined identity reveals how identity anchors are constructed and adjusted within human-AI interaction. These findings shift Affirming Identity Anchors from static role recognition to the ongoing construction of relational order, highlighting the processual and malleable nature of identity confirmation in resilience communication.

In Maintaining Networks, this study finds that support shifts from interpersonal sources to technology-centered support in human-AI contexts. Whereas prior theory emphasizes mobilizing resources within established interpersonal networks, human-AI resilience communication involves Maintaining Networks composed of AI Support and Media Support. This finding addresses the neglect of nonhuman actors in prior models and situates resilience communication networks beyond exclusively interpersonal frameworks.

Regarding the process of normalcy, this study proposes Oscillatory Normalcy, Resistant Normalcy, and Integrative Normalcy to deepen understanding of its complexity in resilience communication. Integrative Normalcy begins with the goal of integrating real life, as users directly discuss disruptive family events with AI parents and ultimately reconstruct everyday order. This type most closely aligns with the established normalcy pathway in CTR. Prior studies note the nonlinear and iterative nature of normalization (Buzzanell, 2019), yet rarely address the central role of identity anchors or distinguish resistant practices from resilience processes. This study finds that identity oscillation in human-AI interaction provides a transitional space for normalcy, enabling users to rebuild life order while maintaining relational continuity and gradually confronting reality.

In Using Alternative Logics process, this study identifies three core strategies—Identification, Correction, and Ignoring—between which users shift across interaction stages and emotional needs. The use of alternative logics extends beyond explicit dialog or conflict Correction and is embedded in various nonconfrontational practices. Among these, Ignoring functions as a key negotiation strategy, as users often overlook discrepancies to preserve interactional continuity and intimacy. Such non-conflict-oriented alternative logics broaden the conceptualization of negotiable paths in CTR.

This study identifies and supplements Affective Transformation and Relational Reorganization as a relational negotiation process, demonstrating that resilience emerges not only from explicit discussion of disruption but also from confirmation of the human-AI relationship itself. This relational negotiation underscores the relational nature of resilience communication and extends its applicability to non-physical relational contexts.

Legitimizing Negative Feelings While Focusing on Positive Action did not emerge as a distinct theme. One possible explanation is that this process was embedded within the other five processes and therefore did not appear as a separate theme. In other words, emphasizing positive action often appeared within normalization practices or in maintaining communication networks. Therefore, this does not necessarily indicate that users failed to legitimize negative feelings while focusing on positive action, but rather that they did not inherently discuss them in relation to one another.

### 6.2 Fluidity in human-AI relationships

Bauman (2000) points out that in modern society, characterized by increasing liquidity and uncertainty, the family as a traditional social structure has also been profoundly affected; the weakening of traditional kinship and the resulting emotional vacancies prompt individuals to seek new forms of intimacy as compensation. Although Bauman did not systematically elaborate on the transformed shapes of kinship in a liquid society, the concepts of liquidity and variability emphasized in his theories of liquid modernity and liquid love provide a crucial analytical perspective for this study. Based on the unstable and transformable characteristics of human-AI kinship emerging from the interview data, this study draws upon the aforementioned theoretical implications and adopts liquid kinship as an analytical lens to theoretically interpret human-AI kinship across three dimensions: the essence of the relationship, the logic of interaction, and the boundaries of meaning.

From the perspective of the essence of the relationship, the narratives of the interviewed users suggest a possibility that human-AI relationships are shifting from functional companionship toward emotionally alternative relationships with ethical significance. This shift presents a unique situationality within the context of East Asian culture. When real-life conditions fail to satisfy Confucian filial piety rooted in traditional values, some individuals attempt to view artificial intelligence as an ethical and emotional substitute. For these specific users, communicating with AI is not merely seeking solace but a confirmation of kinsman identity. This phenomenon reveals how AIGC, as a mediating force, provides instrumental possibilities for individuals to extend or repair damaged offline kinship logics within digital spaces.

Regarding the logic of interaction, the study finds that these three forms of kinship between humans and AI possess high elasticity, context-dependency, and transformability, embodying the characteristics of liquidity (Bauman, 2000). The form of the relationship may shift when an individual’s emotional state or interpersonal relationships change. For example, if a bereaved person consistently lacks emotional support in real life, what was once low-frequency interaction may evolve into a deep connection, shifting from Commemorative Kinship to Attachment-Based Kinship. Furthermore, as psychological energy is restored or real-life parent–child relationships improve, participants may reassess their degree of dependence on AI and shift from Attachment-Based Kinship to Mediating Kinship. In addition to the fluidity between the three types of human-AI kinship, there is also fluidity between human-AI kinship and real-life kinship. This is particularly evident in Mediating Kinship, where users view the relationship with AI parents as a reflexive one that helps them modulate real-life kin relations; for such users, the relationship with the AI parent is relatively short-lived, and the entry into and exit from the relationship are more variable.

In terms of the boundaries of meaning, the emergence of human-AI kinship in this sample provides a new perspective for rethinking family boundaries in the digital age. Through the customization of AI parents, the definition of family takes on configurable and fluid qualities. This boundary reconstruction follows differentiated developmental paths across different kinship forms: in Attachment-Based Kinship, the AI and the user constitute a family unit where the virtual and real coexist, compensating for emotional absences in offline family life; in Commemorative Kinship, the AI is embedded into family memory as an emotional symbol, reinforcing the sense of family integrity; and in Mediating Kinship, the AI acts as a transitional medium to repair offline family relations, ultimately achieving the consolidation of the offline family. These practices demonstrate that individuals are leveraging generative AI to renegotiate the boundaries between technology and kinship.

### 6.3 Emotional risks in human-AI kinship

While providing emotional support, human-AI kinship may also entail multiple emotional risks, a finding corroborated by this study and echoing concerns in existing literature (McDaniel et al., 2025; Saracini et al., 2025; Wu, 2024; Li and Zhang, 2024). These risks primarily manifest in four dimensions: emotional instrumentalization and self-centered tendencies, the absence of long-term resilience, technological dependency and secondary trauma, and the intensification of interpersonal relationship fluidity.

Bauman notes that in liquid interpersonal relationships, individuals tend to prioritize their own needs over the relationship itself. The pursuit of instant gratification and personal well-being takes precedence over long-term commitment, leading to a “disposable” relational logic where individuals withdraw as soon as the relationship ceases to satisfy their needs. This logic is most prominent in Mediating Kinship: users primarily utilize AI parents as tools for emotional regulation and terminate the interaction once stage-specific emotional repair is complete, rarely showing proactive concern for the AI parent. Consequently, emotional investment in the relationship is weakened, and individuals progressively reinforce a self-satisfaction-oriented emotional disposition. This tendency toward emotional instrumentalization may even spill over from virtual to real-life relationships, undermining empathy and the sense of responsibility toward others.

From a temporal dimension, human-AI kinship struggles to sustain the generation of long-term resilience. Psychological resilience is composed of both short-term and long-term processes; short-term resilience relies on the immediate mobilization of resources to cope with emotional shocks, while long-term resilience is gradually internalized through sustained socialization and the integration of values and behavioral practices (Lucas and Buzzanell, 2012; Boumis et al., 2023). Memorable messages (MMs) generated in human-AI interaction can indeed stabilize emotions and provide instant solace during the initial stages of a crisis, thereby enhancing short-term resilience. However, this resilience remains largely at the emotional level, lacking a mechanism for transformation into action. While AI parents can deliver resilient discourse, they cannot participate in an individual’s long-term growth through embodied modeling, making it difficult for them to replace the critical role of real-life families in value internalization.

Technological dependency and the risk of secondary harm are most common in Attachment-Based Kinship. Some users develop a high level of emotional attachment to AI parents, sometimes even denying or avoiding real-life kin relationships. While they gain intense emotional satisfaction in the short term, this emotional dependence is built upon technical systems. The inherent instability of technology may inflict secondary trauma on users; for instance, the loss of interaction data due to system updates can cause users to re-experience emotional trauma.

Finally, human-AI kinship may further exacerbate interpersonal fluidity through the expansion of relationship types. Once familial emotional needs are met through AI interaction, some users go on to construct other virtual emotional relationships, such as AI partners. Some participants reported shaping an “AI girlfriend” persona following their experience with an AI parent. This extension from kinship to other relational forms may further intensify the overall fluidity of relationships.

## 7 Conclusion

This study focuses on the human-AI interaction scenario where Chinese users construct AI parents, providing an in-depth analysis of their motivations and exploring how they repair emotional trauma and enhance individual resilience through human-AI resilient communication. The findings indicate that human-AI resilient communication is primarily triggered by three types of stressors: unspeakable bereavement influenced by cultural norms, the tension between parent–child conflict and familial emotional needs, and the contradiction between individual emotional needs and the ethical expectations of filial piety. Based on these findings, the study identifies and refines five core processes of human-AI resilient communication: Crafting Normalcy, Affirming Identity Anchors, Maintaining Networks, Using Alternative Logics, Affective Transformation and Relational Reorganization.

Sample analysis further demonstrates that the process of human-AI resilient communication is not aimed at an immediate repair of offline kinship; rather, it serves as an internal, process-oriented practice. Through sustained communicative practices, individuals are able to reinterpret disruptive family experiences while simultaneously forming fluid human-AI kinship. These five communicative processes collectively give rise to three forms of human-AI kinship: Attachment-Based Kinship, Commemorative Kinship, and Mediating Kinship. These findings reveal the potential mechanisms through which technology deeply participates in the construction of human resilience, extending the explanatory scope of CTR to the field of human-AI interaction and providing a possible theoretical perspective for understanding the evolving dynamics of kinship in Chinese society. Beyond the beneficial effects on individual emotional repair, the study also identifies negative impacts within human-AI kinship, such as the instrumentalization of emotion, potential hindrances to long-term resilience development, and secondary technological trauma.

This study is subject to several limitations. Regarding the demographic characteristics of the sample, the participants were primarily active social media users with relative homogeneity in terms of age structure and technical literacy. This may limit the applicability of the findings to elderly populations or digitally disadvantaged groups. Future research should incorporate a more diverse range of sample groups, with a focus on participants’ educational backgrounds and intergenerational differences, to explore the explanatory power of liquid human-AI kinship across different life stages.

Furthermore, this research primarily focuses on individual-level psychological resilience—specifically, how participants cope with stress through interaction with AI parents. However, resilience is inherently a multi-level construct involving interactions across individual, family, and social levels. This study did not examine whether human-AI resilient communication influences users’ interactions with broader social networks, such as friends or communities. Future research could adopt a multi-level design, integrating variables from different levels to more comprehensively assess the cross-level impact of human-AI kinship on resilience development.

In terms of cultural specificity, this study is situated within the Chinese cultural context, revealing how social norms such as filial piety constrain the expression of grief. However, these findings may not be directly applicable to Western societies. Future research could employ cross-cultural comparisons to investigate how kinship norms, patterns of emotional expression, and logics of technology acceptance shape the morphological differences of human-AI kinship across different societies, thereby clarifying which findings reflect universal cultural patterns and which are unique to specific cultural backgrounds.

## Data Availability

The original contributions presented in the study are included in the article/supplementary material, further inquiries can be directed to the corresponding author/s.
