# Correction: ‘She lifted me up': kinship construction in human-AI resilient communication among Chinese users

**DOI:** 10.3389/fpsyg.2026.1937561

**Published:** 2026-07-24

**Authors:** Frontiers Production Office

**Affiliations:** Frontiers Media SA, Lausanne, Switzerland

**Keywords:** AI parents, communication theory of resilience, human-machine communication, liquid kinship, human-AI relationships

A paragraph that was intended for removal from the manuscript was inadvertently retained during the production process.

A correction has been made to the Section **Introduction**, paragraph 2. This paragraph has now been deleted:

“Beyond common AI roles such as friends or romantic partners, parents—a role traditionally grounded in blood ties, responsibility, and emotional authority—have increasingly become objects of AI-mediated emotional engagement (Pillay, 2025). The rise of AI parents has renewed public concerns about artificial intelligence use, including parental identity anxiety—“If she is my daughter's mother, then who am I?”—and children's safety concerns, such as whether family privacy may be technologically repurposed. These controversies suggest that generating parental figures through AI is not merely a technical application but implicates social norms of kinship, boundaries of identity, and established emotional orders.”

The original version of this article has been updated.

